# WHO-Point prevalence survey of antibiotic use in a Senegalese tertiary care hospital

**DOI:** 10.1371/journal.pgph.0004887

**Published:** 2025-07-21

**Authors:** Moustapha Diop, Fatimata Wone, Oumar Bassoum, Mariéme Ndiaye, Adji Marème Diagne, Tracie Youbong, Sokhna Moumi Mbacké Daffé, Maguette Ndoye, Ajuamendem Ghogomu Tamouh, Mamadou Wagué Gueye, Mor Ngom, Bécaye Fall, Papa Samba Ba, Adama Faye

**Affiliations:** 1 Department of Infectious and Tropical Diseases, Dakar Principal Hospital, Dakar, Senegal; 2 Department of Infectious and Tropical Diseases, Dalal JAMM Hospital, Dakar, Senegal; 3 Institute of Health and Development, Cheikh Anta Diop University of Dakar, Dakar, Senegal; 4 University of Health Sciences of Dakar, Dakar, Senegal; 5 Federation of Laboratories, Dakar Principal Hospital, Dakar, Senegal; 6 Department of Human Resources, Ministry of Public Health, Yaounde, Cameroon; 7 Laboratory Directorate, Ministry of Health and Social Action, Dakar, Senegal; Washington State University, UNITED STATES OF AMERICA

## Abstract

The inappropriate use of antibiotics in human medicine is one of the primary causes of antimicrobial resistance. The objective of this study was to estimate the prevalence of antibiotic use at Dakar Principal Hospital and to identify factors associated with the prescription of broad-spectrum antibiotics. This cross-sectional study, conducted according to the WHO-point prevalence survey method, included all patients hospitalized in acute care wards who presented at 8:00 AM on the day of the survey. Data were collected from 9 to 29 December 2024 on working days. Multivariate logistic regression was performed to identify factors associated with the prescription of broad-spectrum antibiotics. A total of 222 patients, predominantly male (sex ratio = 1.26), were included. The median age of patients over 2 years was 45 years (interquartile range: 29–64 years). In total, 158 antibiotic prescriptions were reported for 101 included patients, resulting in a prevalence of 45.5% (101/222) and a prescription ratio of 1.56 antibiotics per patient. The most commonly prescribed antibiotics were amoxicillin-clavulanic acid (n = 36; 16.2%), followed by ceftriaxone (n = 21; 9.5%). Community acquired infection was the most common reason for prescription (n = 86; 54.4%), and 98 prescriptions (62%) were in compliance with the local guidelines. Antibiotics from the watch group of the AWaRe classification were used in 55 patients (54.4%). According to the multivariate analysis, the presence of a healthcare-associated infection was associated with this use (OR = 12.1; 95% CI [2.62–93.7]). These antibiotics from the watch group were significantly less commonly prescribed for surgical prophylaxis (OR = 0.13; 95% CI [0.02 - 0.63]). The prevalence of antibiotic use was high in the studied facility, with more prescriptions belonging to the watch group. These results underscore the need to strengthen antimicrobial stewardship policies.

## 1. Introduction

Antimicrobial resistance (AMR) is a global public health problem. In the absence of adequate measures by 2050, approximately 10 million deaths per year and significant economic consequences due to AMR are expected [1]. In 2019, the number of deaths associated with bacterial resistance to antibiotics worldwide was estimated at 4.95 million, with 1.27 million deaths directly attributable to this phenomenon [2]. The burden of bacterial resistance to antibiotics is particularly concerning in Sub-Saharan Africa. According to a recent meta-analysis conducted in the West African region, 59% of the bacteria tested were multidrug resistant, with a 7% increase from 2010--2024 [3]. The death rate related to infections caused by resistant bacteria reported in Sub-Saharan Africa in 2019 was estimated at 27.3 deaths per 100,000 inhabitants [2]. The WHO estimates that the misuse of antibiotics in human, animal, and plant health is the main factor leading to the emergence of MDR bacteria [4]. This is the reason why appropriate use of antimicrobials represents the fourth goal of the global plan to fight against antimicrobial resistance [5]. As a result, a standardized protocol has been developed for conducting point prevalence surveys (PPSs) on a single day to better assess antibiotic use by prescribers in healthcare facilities [6]. The goal of PPSs is to perform a situational analysis of antibiotic prescriptions in a hospital to help set up and run an antimicrobial stewardship program. It also helps to monitor the prescription of antibiotics in healthcare facilities. Several PPSs on antibiotic use have been conducted in African countries in recent years. A systematic review including 28 PPSs in sub-Saharan African countries estimated that the prevalence of antibiotic use was 64%, with a significant predominance in intensive care units (ICU) (89%), and the most commonly prescribed antibiotics were ceftriaxone, metronidazole, and gentamicin [7]. However, few PPSs have been conducted in West Africa. In Senegal, data on antibiotic prescriptions derived from the PPS, as recommended by the WHO, are lacking in the literature. Previous studies on antibiotic use have focused on patients treated in outpatient settings in primary healthcare services or in university hospitals [8–10]. Therefore, we conducted this study, which aimed to estimate the prevalence of antibiotic use in hospitalized patients at Dakar Principal Hospital on the day of the survey and to evaluate the factors associated with the use of broad-spectrum antibiotics.

## 2. Study setting

Dakar Principal Hospital was established in 1884 and was designated a military training hospital in 2008 by Decree No. 2008--1001 dated August 18, 2008.

Serving as a referral facility for the Senegalese armed forces, it is a tertiary care hospital that operates within the South Healthcare District of Dakar. Dakar Principal Hospital has a bed capacity of 372, with a total of 220 healthcare providers.

In 2024, the hospital admitted 13,590 patients, with an average length of stay of 6.7 days. During the same period, 872 deaths were recorded, corresponding to a mortality rate of 6.4%. The bed turnover rate and average bed occupancy rate were 36.5% and 66.7%, respectively. Dakar Principal Hospital has two primary missions:

- Public Service Missions: As a tertiary healthcare institution, its goal is to ensure equitable access to healthcare services for all citizens.
- Specific Missions for the Armed Forces: This encompasses expertise in tropical medicine, the establishment of a humanitarian reception and emergency training platform, continuous readiness for exceptional civilian and military situations in West Africa, and serving as a training center and support hub for the Armed Forces.

**Fig 1:**
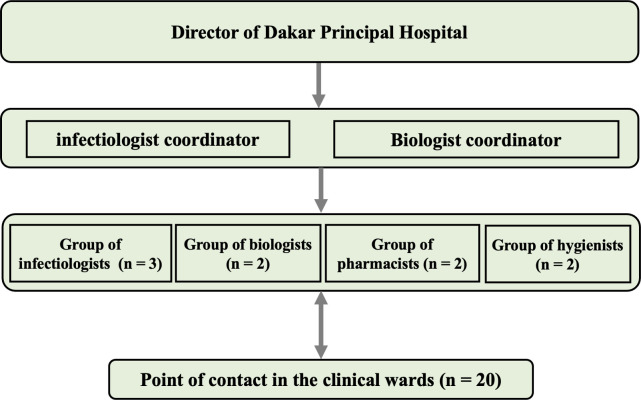
Organizational chart of the mobile team.

Dakar Principal Hospital also has had a mobile multidisciplinary antibiotic team (MAT) since July 2023, which gathers with the Infectious and Tropical Diseases Department, the laboratories ward, the hospital pharmacy, and the infection prevention and control team. The MAT was the first in West Africa. The objective of the MAT is to ensure optimal patient care by promoting the appropriate use of antibiotics across all clinical departments. The members of the MAT are presented in Fig 1.

The activities of the mobile team are as follows:

- Antibiotic therapy advice, provided for any requesting department or following positive sample reports from the laboratory.
- Efficient management of antibiotics, which is the primary role of hospital pharmacists in collaboration with other MAT members.
- Training and awareness sessions for medical and paramedical staff on the proper use of antibiotics, microbiological sampling, and infection prevention and control measures.
- Periodic evaluation of MAT activities on the basis of objective and measurable performance indicators.
- An antibiotic therapy guide writing based on current recommendations from scientific societies and adapted to the hospital’s local context

## 3. Methodology

### 3.1 Study design and period

This was a descriptive cross-sectional study based on the WHO PPS protocol of antibiotic use [6] from 9 to 29 December 2024 on working days with an analytical component.

### 3.2 Study population

The study population consisted of patients hospitalized at Dakar Principal Hospital during the study period. Those who were present at 8 a.m. on the day of the survey in acute care wards were included. All newborns born before 8 a.m. on the day of the survey, as well as their mothers, were included and counted separately. Patients who were not included were those who were unavailable or had unusable medical records on the day of the survey or those who were hospitalized in:

- Long-term care units
- The emergency department
- Outpatient care wards

### 3.3 Sampling

Dakar Principal Hospital has fewer than 500 hospital beds. In such a situation, the WHO recommends recruiting all patients meeting the inclusion criteria. Consequently, the sample size was not calculated, and the sampling was exhaustive.

### 3.4 Definition of terms

#### 3.4.1 Antibiotic use.

A patient was considered to receive antibiotics if:

- The patient was receiving antibiotics at 8:00 AM on the day of the survey.
- The antibiotics administered are listed in the WHO PPS protocol [5].
- The antibiotic was administered via oral, parenteral, rectal, or inhalation routes.
- The antibiotic was administered within 24 hours prior to 8:00 AM on the day of the survey in the case of a single dose.

Patients receiving topical or ophthalmic antibiotics or suppositories and those with an antibiotic regimen started after 8:00 AM or interrupted before 8:00 AM were not considered to receive antibiotics.

#### 3.4.2 Healthcare-associated infections.

An infection is classified as healthcare-associated infection if the onset occurs:

- From the third day following admission, OR
- On the first or second day after admission AND the patient was transferred from another hospital, OR
- On the first or second day after admission AND the patient was discharged from a hospital (the same or another) within the previous 48 hours before the new admission.

#### 3.4.3 *AWaRe* classification.

The *AWaRe* classification is a WHO classification that groups antibiotics into three categories on the basis of their spectrum of activity and their potential risk of inducing bacterial resistance [11]

### 3.5 Calculation of antibiotic doses

The daily dose of antibiotics was calculated by multiplying the unit dose by the daily frequency of administration. When the antibiotic was administered every n.h. (n > 24 hours), the daily frequency was 24/n. Thus, the daily dose of the antibiotic would be equal to the unit dose × 24/n.

When the dose was expressed in mg (or IU) per kg per day for patients over 13 years old, the daily dose in mg (or IU) was calculated using a standard weight of 70 kg, regardless of the patient’s actual weight. For children under 13 years of age, the daily dose was calculated in mg (or IU) on the basis of the actual weight of the patient.

The dose of the combined products corresponded to the sum of the antibiotic substances in the combination, excluding the enzyme inhibitors.

### 3.6 Data collection

#### 3.6.1 Data source.

Data regarding the services were collected from the administrative units of the hospital. Patient data were gathered from medical records, other written sources, and hospital staff as a last resort. Structured individual interviews were also conducted with patients or their legal guardians, if needed, to obtain additional information.

#### 3.6.2 Data collection tools and procedures.

The standardized WHO questionnaire was used for data collection, which was then transformed into an electronic questionnaire via kobocollect. It was pretested on 15 patients from different departments before the start of the survey. Two investigators were trained on the protocol and questionnaire. One survey day was dedicated to each department, and data collection was completed for the entire hospital within three consecutive weeks from the first day of data collection.

Data related to the services, including patients (sociodemographic characteristics, comorbidities), indications for antibiotic therapy, and prescribed antibiotics (number, names and families of antibiotics, unit of measurement, route and frequency of administration, daily doses, quality of the prescription), were collected.

### 3.7 Statistical data analysis

#### 3.7.1 Description of variables.

According to nature of the variables, they were expressed as the means ± standard deviations, medians and interquartile ranges (IQR) or numbers and proportions. The prevalence of antibiotic use in patients was a ratio, with the numerator being the number of patients receiving a least one antibiotic at 8:00 AM and the denominator being the total number of hospitalized patients present at 8:00 AM on the day of the survey.

#### 3.7.2 Factors associated with the use of broad-spectrum antibiotics.

The binary variable “use of broad-spectrum antibiotics,” corresponding to an antibiotic belonging to the watch or reserve groups in the AWaRe classification [11], was considered the variable of interest with categories “Yes” or “No.” To compare the means of potentially explanatory quantitative variables, we used Student’s t test or the Wilcoxon rank-sum test, depending on applicability. To compare proportions of potentially explanatory qualitative variables, we used Pearson’s chi-square test, Yates’ corrected chi-square test, or Fisher’s test, depending on applicability. A multivariate logistic regression was then performed, including all explanatory variables with p-values less than 0.2 in the bivariate analysis. The Hosmer–Lemeshow goodness-of-fit test was conducted to assess the model’s calibration. The leverage effect was examined graphically, and individuals deviating from the population were removed from the dataset. Interaction terms between variables were also examined. At the end of this analysis, the association between the variable of interest and explanatory variables was considered statistically significant when the 95% CI for the adjusted odds ratio excluded 1. The data were analyzed via R version 4.3.3 (2024-02-29).

### 3.8 Mitigation of potential biases

To ensure the internal validity of our study, several measures were implemented to minimize methodological biases.

- Management of selection bias

All admitted patients in the acute care wards of the hospital, present at 8 a.m. on the day of the survey, were included, enabling a representative sample. The data were collected at one single day, which helped minimize missing data. Data collection was also conducted in working days using clearly defined inclusion and non-inclusion criteria to enroll as many eligible patients as possible.

- Management of information bias

A standardized case report form inspired by the WHO protocol was used for data collection. two trained investigators on the questionnaire were in charge to collect information. The tool was pilot-tested on 15 patients from three different departments (pediatrics, intensive care unit, and visceral surgery), leading to revisions in the wording and structure of some questions. Daily debriefings were held to supervise data collection and make necessary corrections.

• Management of confounding bias

A multivariate logistic regression analysis was conducted to adjust for potential confounding factors

### 3.9 Ethical considerations

Prior to the study, written authorization was requested from the hospital’s director. The study also received ethical approval from the National Ethics Committee for Health Research of Senegal (Number: 0000197 MSAS/CNERS/SP).

it was conducted in accordance with the revised version of the Declaration of Helsinki and applicable principles of the International Committee on Harmonization of Good Clinical Practice. The confidentiality of the information collected and the anonymity of the participants were respected, and the data were stored in a secure database. Informed consent was obtained from the participants or their legal guardians before inclusion in the survey. No additional physical or paraclinical examinations were performed by the research team. The risk associated with implementing this survey was considered minimal in terms of physical, emotional, psychological, legal, social, or economic impact.

## 4. Results

### 4.1 Selection and distribution of the study population

**Fig 2:**
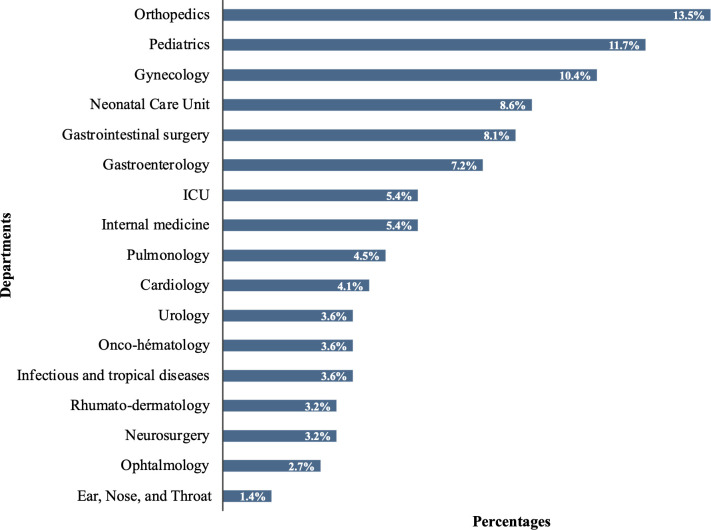
Presentation of the different wards included on the day of the survey (N = 17).

Among the 227 eligible patients in the 17 targeted departments at 08:00 on the survey day, 222 (97.8%) were included. Among them, 95 (42.8%) were hospitalized in the surgery department, 70 (31.5%) were hospitalized in the medical department, 45 (20.3%) were hospitalized in pediatrics, and 12 (5.4%) were hospitalized in the intensive care unit. The most represented patients were from orthopedics (n = 30; 13.5%), pediatrics (n = 26; 11.7%), and gynecology (n = 23; 10.4%) (Fig 2).

### 4.2 Baseline characteristics of the study population

Men were more represented, with a sex ratio of 1.26. Patients over 2 years of age accounted for 85.6% of the study population, with a median age of 45 years (interquartile range: 29–64 years). Newborns, representing 13% of the study population, had a median age of 3 days (interquartile range: 1–12 days). The three children under 23 months of age were aged 1 month, 2 months, and 12 months.

For newborns, the gestational age, specified for 10 patients, ranged between 37 and 38 weeks in half of the cases. The birth weight, which was specified for 25 patients, ranged between 0.67 and 2.5 kg in 64% of the patients. Sixty-six patients (29.7%) had at least one comorbidity. Hypertension and diabetes were the most common, with proportions of 13% and 12%, respectively. The baseline characteristics of the study population are presented in Table 1.

**Table 1:** Baseline characteristics of the study population on the day of the survey (n = 222).

Characteristics	Number	Percentages
Age group		
≤ 1 month	29	13%
Between 1 and 23 months	3	1.4%
≥ 2 years	190	85.6%
Sex		
Male	124	56%
Female	98	44%
Gestational age in GW (n = 10)		
37 – 38	5	50%
39 – 40	1	10%
≥ 41	4	40%
Birth weight in kg (n = 25)		
[0.67 - 2.5]	16	64%
[2.5 - 3.5]	7	28%
[3.5 - 4.115]	2	8%
Comorbidities		
Diabetes	26	12%
Hypertension	29	13%
Heart disease	15	6.8%
Recent chemotherapy less than 3 months	12	5.4%
Chronic pulmonary diseases	8	3.6%
HIV infection	4	1.8%
Sickle cell disease	6	2.7%
Chronic neuropsychiatric disorders	5	2.3%
Congenital diseases	5	2.3%
Chronic gastroenterological diseases	3	1.4%
Glaucoma	3	1.4%
Preterm birth	3	1.4%
Autoimmune disease	3	1.4%
Chronic kidney diseases	2	0.9%
Osteoarthritis	2	0.9%
Dyslipidemia	2	0.9%
Hypothyroidism	2	0.9%
Prostatic diseases	2	0.9%
Hyperuricemia	1	0.5%
Traumatism	1	0.5%
Hepatitis B	1	0.5%

### 4.3 Prevalence of antibiotic use among patients

**Fig 3:**
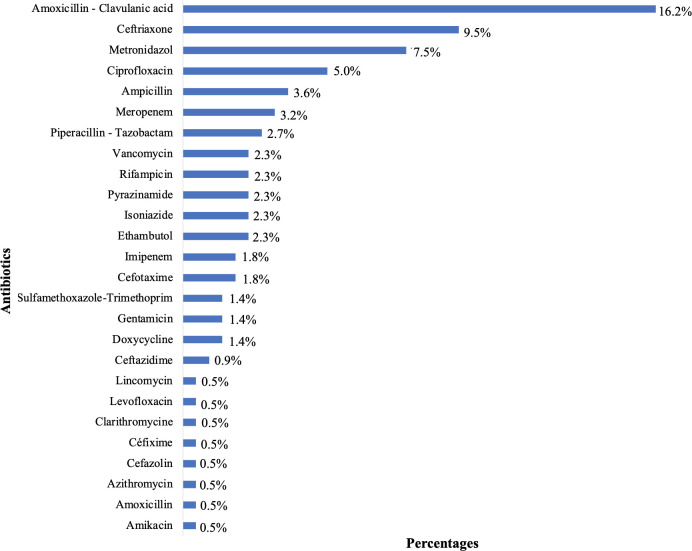
Distribution of the prevalence of antibiotics prescribed on the day of the survey (N = 222).

Among the 222 included patients, 101 were receiving at least one antibiotic, representing a point prevalence of 45.5%. These 101 patients received a total of 158 antibiotic prescriptions, resulting in a ratio of 1.56 antibiotics per patient. The beta-lactam class was the most commonly used (n = 91; 90.1%), particularly amoxicillin-clavulanic acid (n = 36; 16.2%) and ceftriaxone (n = 21; 9.5%), followed by imidazoles (n = 17; 7.5%) (Fig 3). The majority of patients were on a single antibiotic (58.4%), while 5 patients (5.0%) were receiving four antibiotics.

### 4.4 Distribution of prescriptions by department

**Fig 4:**
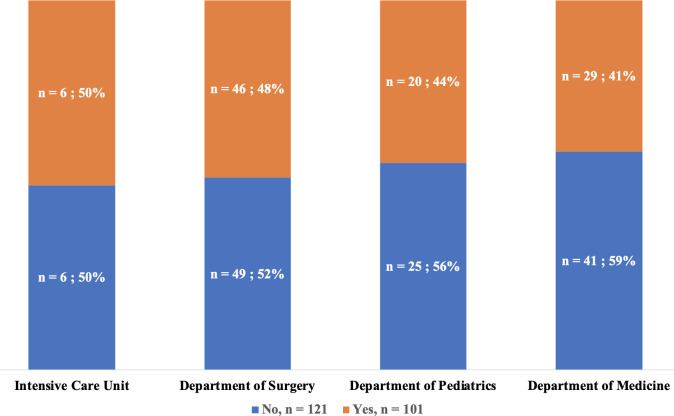
Distribution of the prevalence of antibiotic use by department on the day of the survey (N = 222).

**Fig 5:**
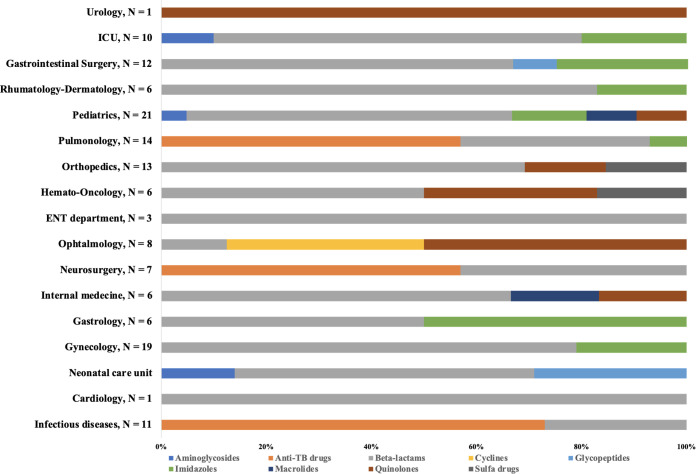
Distribution of families of antibiotics by ward on the day of the survey (N = 158).

Antibiotic use was most common in the intensive care unit, where half of the patients were on antibiotics (6/12) (Fig 4). Beta-lactams were used in 94.1% of the included departments (16/17) and accounted for 100% of prescriptions in cardiology and Ear, Nose, and Throat (ENT) departments. Imidazoles and quinolones were used in 7 (41.2%) and 6 (35.3%) of the included departments, respectively. Anti-tuberculosis drugs (anti-TB drugs) were the most commonly prescribed antibiotic class for infectious diseases, pulmonology, and neurosurgery, representing 73%, 57%, and 57%, respectively, of all prescriptions in these departments (Fig 5). Tetracyclines were the least commonly used class and were prescribed only in ophthalmology, where they accounted for 37.5% of all prescriptions in that department.

### 4.5 Description of antibiotic therapy among patients

Antibiotic therapy protocols were based on at least one biomarker in 56 patients (55.4%), with the most commonly used being C-reactive protein (CRP) (85.7%) and white blood cell count (83.9%). For 46 patients (45.5%), a sample was sent for bacteriological documentation. Blood, pus, and urine were the most frequently collected samples, representing 67.4%, 26.1%, and 8.7%, respectively (Table 2).

**Table 2:** Characteristics of antibiotic therapy protocols among patients on the day of the survey (n = 101).

Characteristic	Number	Percentages
Number of antibiotics		
1	59	58.3%
2	32	31.7%
3	5	5.0%
4	5	5.0%
Biomarkers (n = 56)		
CRP	48	85.7%
White blood cells	47	83.9%
Procalcitonin	1	1.8%
Cephalo-spinal fluid cytochemistry	1	1.8%
Samples for documentation (n = 46)		
Blood	31	67.4%
Pus	12	26.1%
Urine	4	8.7%
Cerebrospinal fluid	2	4.3%
Bronchoalveolar lavage fluid	2	4.3%
Gastric tubing	2	4.3%
Pleural fluid	2	4.3%
Stools	1	2.2%
Sputum	1	2.2%
Catheter tip	1	2.2%
Peritoneal fluid	1	2.2%
Gastric biopsy puncture	1	2.2%
Bronchial secretion	1	2.2%

### 4.6 Description of the antibiotic prescriptions

Among the 158 antibiotic prescriptions, 124 (78.5%) were for curative purposes, of which 91 (73.4%) were not documented. The majority of antibiotic prescriptions (n = 148; 94%) were for a single indication. Community-acquired infections were the most common reason for prescription (n = 86; 54.4%), followed by healthcare-associated infections (n = 38; 24.1%). Surgical antibiotic prophylaxis accounted for 16.5% of prescriptions, with multiple doses beyond one day in 18 cases (69.2%) and multiple doses within a single day in 8 cases (30.8%). The reason for antibiotic therapy was unknown for 5 prescriptions (3.2%). The main indications for curative antibiotic therapy were tuberculosis (20; 12.7%), lower respiratory tract infections (19; 12%), skin and soft tissue infections (16; 10.1%), and neonatal infections (14; 8.9%).

The different indications for surgical antibiotic prophylaxis (n = 26) included gynecological-obstetric surgery (n = 16; 61.5%), orthopedic surgery (n = 4; 15.4%), ocular surgery (n = 3; 11.5%), neurosurgery (n = 2; 7.7%), and ENT surgery (n = 1; 3.8%). With respect to the quality of prescriptions, in more than two-thirds of prescriptions, the start date, indication, and international nonproprietary name of the prescribed antibiotic were not mentioned in the medical records, and 37 prescriptions (23%) did not comply with the guidelines for proper use. A description of the antibiotic prescriptions is presented in Table 3.

**Table 3:** Characteristics of the antibiotic prescriptions on the day of the survey (N = 158).

Characteristic	Number	Percentages
Start date specified in the file	137	86.7%
Written indication in the file	97	61.4%
the International Nonproprietary Name mentioned in the file	24	15.2%
Type of antibiotics		
Combined	3	1.9%
Unique	155	98.1%
Unit of measurement		
g	62	39%
mg	96	61%
IU	0	N/A
Frequency of administration (per day)		
1	45	28.5%
2	43	27.2%
3	68	43%
4	2	1.3%
Daily dose in mg (median and IQR)	1,500	525 - 2750
Route of administration		
Parenteral	106	67%
Oral	52	33%
Number of indications		
0	5	3.2%
1	148	93.7%
2	4	2.5%
3	1	0.6%
Existence of missed doses	2	1.3%
Number of missed doses		
1	1	50%
4	1	50%
Curative antibiotic therapy type (n = 124)		
Probabilistic	91	73.4%
documented	33	26.6%
Types of indications		
Community acquired infection	86	54.4%
Hospital acquired infections	38	24.1%
Surgical prophylaxis	26	16.5%
Medical prophylaxis	8	5.1%
Unknown	5	3.2%
Compliance with good practice guidelines		
No	37	23%
Yes	98	62%
No information	19	12%
No recommendation	4	3%
Indications		
Tuberculosis	20	12.7%
Lower respiratory infection	19	12.0%
Prophylaxis in gynecology surgery	16	10.1%
Soft tissue and skin infection	16	10.1%
Neonatal infection	14	8.9%
Gastrointestinal surgery infection	12	7.6%
Join and bone infection	11	7.0%
Unknown	9	5.7%
Gastrointestinal infection	9	5.7%
Medical prophylaxis	8	5.1%
Lower urinary tract infection	6	3.8%
Bacteriemia with unknown entry	5	3.2%
Central nervous system infection	5	3.2%
Eyes infection	5	3.2%
Prophylaxis in orthopedic surgery	4	2.5%
Prophylaxis in eyes surgery	3	1.9%
Prophylaxis in central nervous system surgery	2	1.3%
Prophylaxis in ENT surgery	1	0.6%
ENT infection	1	0.6%
Prophylaxis in gastrointestinal surgery	1	0.6%
Upper urinary tract infection	1	0.6%

### 4.7 AWaRe classification

#### 4.7.1 Use of different antibiotic classes (Fig 6).

Antibiotics from the watch group of the AWaRe classification were used in 55 patients (54.4%) and accounted for 40.5% of antibiotic prescriptions (64/158). Antibiotics from the access group were prescribed to 41 patients (40.6%), representing 46.8% of the prescriptions (74/158). The remaining 5 patients (5%) were prescribed nonclassified antibiotics (NCs), accounting for 20 prescriptions (12.7%). No antibiotics from the reserve group were used in our study population. All six newborns in the nursery receiving antibiotics were given antibiotics from the watch group. There was also significant use of antibiotics from the watch group in visceral surgery (87.5%), intensive care (83.3%), and pediatric care (64.2%). Antibiotics from the access group were more commonly used in gynecology (85.7%) and orthopedics (63.6%).

**Fig 6:**
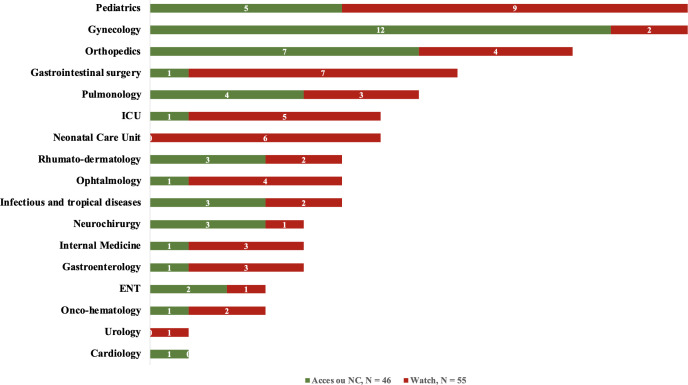
Distribution of different categories of AWaRe classification by ward on the day of the survey (n = 101).

#### 4.7.2 Factors associated with the use of antibiotics from watch class.

According to the univariate analysis, male sex (p = 0.048), healthcare-associated infections (p < 0.001), and microbiological documentation of infections (p = 0.017) were significantly associated with the use of antibiotics in the watch group (Table 4). This group of antibiotics was significantly less commonly used in surgical prophylaxis (p < 0.001).

**Table 4:** Bivariate assessment of factors associated with watch group use on the day of the survey (N = 101).

	Antibiotics from watch group		
Characteristic	Yes, N = 55 ^*1*^	No, N = 46 ^*1*^	p
Age groups			0.4
≤ 1 month	7 (78%)	2 (22%)	
between 1 and 23 months	2 (67%)	1 (33%)	
≥ 2 years	46 (52%)	43 (48%)	
Sex			0.048
Female	18 (43%)	24 (57%)	
Male	37 (63%)	22 (37%)	
Surgical prophylaxis			<0.001
Yes	3 (14%)	19 (86%)	
No	52 (66%)	27 (34%)	
Medical prophylaxis			>0.9
Yes	3 (60%)	2 (40%)	
No	52 (54%)	44 (46%)	
healthcare-associated infections			<0.001
Yes	25 (93%)	2 (7%)	
No	30 (41%)	44 (59%)	
Documented Infection			0.017
Yes	15 (79%)	4 (21%)	
No	40 (49%)	42 (51%)	
Department			0.054
Surgery	20 (43%)	26 (57%)	
Medicine	15 (52%)	14 (48%)	
Pediatrics	15 (75%)	5 (25%)	
ICU	5 (83%)	1 (17%)	
Comorbidity			0.2
Yes	33 (50%)	33 (50%)	
No	22 (63%)	13 (37%)	

After adjustment for potential confounding factors through multivariate logistic regression, antibiotics from the Watch group were 12 times more commonly used in healthcare-associated infections and 7.7 times less commonly prescribed in surgical prophylaxis (Table 5).

**Table 5:** Multivariate assessment of factors associated with watch group use on the day of the survey (N = 101).

Characteristics	OR^1^	95% CI^1^	p
Sex			
Female	—	—	
Male	1.08	0.36, 3.14	0.9
Age groups			
≤ 1 month	—	—	
Between 1 and 23 months	0.22	0.01, 6.74	0.4
≥ 2 years	1.98	0.17, 23.26	0.6
healthcare-associated infections			
No	—	—	
Yes	12.1	2.62, 93.7	0.004
Surgical prophylaxis			
No	—	—	
Yes	0.13	0.02, 0.63	0.016
Documented infection			
No	—	—	
Yes	1.41	0.28, 7.34	0.7
Department			
Surgery	—	—	
Medicine	0.53	0.14, 1.96	0.3
Pediatrics	2.17	0.33, 19.4	0.4
ICU	1.91	0.18, 45.5	0.6
Comorbidity			
No	—	—	
Yes	0.50	0.16, 1.52	0.2

^1^ *OR = Odds Ratio, CI = Confidence Interval, AIC = 108; Hoslem.test: p < 0.05*

## 5. Discussion

This cross-sectional point prevalence survey revealed relatively frequent use of antibiotics, particularly amoxicillin-clavulanic acid, ceftriaxone, and metronidazole, in the study setting. The quality of antibiotic prescriptions for hospitalized patients was also assessed. Multivariate analysis revealed that antibiotics from the Watch group were significantly more commonly used for healthcare-associated infections and less commonly used for surgical antibiotic prophylaxis.

### 5.1 Point prevalence

The point prevalence of antibiotic use in this study was 45.5%. This percentage is lower than that reported in a systematic review in sub-Saharan Africa, which was 64% [7]. It was also lower than the prevalence reported by many other surveys conducted in West African countries, such as Benin (82.9%) [12], Nigeria (80.1%) [13], Sierra Leone (73.7%) [14], Ghana (60.5%) [15], and Niger (54.5%) [16]. Similarly, it was lower than the prevalence reported in surveys from Maghreb countries such as Egypt (59%) [17] and Europe, such as Italy (87.5%) [18]. However, the prevalence reported in this study was higher than that reported in a multicentric Ethiopian survey, which reported a prevalence of 40.3% [19]. Surveys conducted in France in 2010, including 314 hospitals [20], and in China in 2020 [21] reported significantly lower prevalences (19.5% and 28.2%, respectively). These discrepancies could be related to differences in the levels of care in the included healthcare facilities. Antibiotic usage policies, which vary from country to country and from one healthcare facility to another, could also explain this. The existence of a multidisciplinary antibiotic stewardship team at Dakar Principal Hospital, providing advice on antibiotic therapy in clinical departments and training prescribers, could partially explain the less frequent use of antibiotics in this facility than in other hospitals in sub-Saharan Africa.

### 5.2 Predominance in the ICU

In the present study, antibiotics were more frequently used in the intensive care unit, where one in two patients received at least one antibiotic. A similar result was reported in the meta-analysis by Boltena MT et al. [7], who reported more frequent use of antibiotics in intensive care units. A multicentric survey by Omoulo et al. in Kenya also revealed that the prevalence of antibiotic use was significantly higher in the ICU across all three public hospitals included [22]. However, a study by Skosana et al. in South Africa in 2022 reported different results, with a lower prevalence of antibiotic use in the ICU (15.7%) than in medical wards (74.7%) [23]. These differences could be related to the specificity of the healthcare settings and patients included or the one-day design of the survey. It is nonetheless common to observe overuse of antibiotics in ICUs due to the immunocompromised state and the seriousness of the diseases managed in intensive care units, which promotes the occurrence of bacterial infections and, thus, increased antibiotic use. Additionally, the higher frequency of healthcare-associated infections with multidrug resistant bacteria in ICUs due to invasive procedures may also explain the more frequent use of antibiotics [24–26].

### 5.3 Most commonly used antibiotics

The prevalence of beta-lactam use among the included patients was significantly greater in this study, accounting for 90.1%. Amoxicillin-clavulanic acid and ceftriaxone were the two most commonly used beta-lactams, with metronidazole, an imidazole, ranking third. These results corroborate most African and other regional cross-sectional prevalence surveys. Tapha et al. in Niger reported more frequent use of third-generation cephalosporins (48.7%), imidazoles (16.5%), and broad-spectrum penicillins (10.8%) [16]. Similarly, a multicentric survey in Egypt revealed that third-generation cephalosporins were the most commonly prescribed antibiotics, with a prevalence of 28.4%, followed by penicillins with inhibitors and imidazoles at 19.7% and 15.2%, respectively [17]. In a survey by Aboderin et al. in Nigeria, metronidazole (25.2%), cefuroxime (18.4%), and ceftriaxone (77/564, 13.7%) were the most commonly prescribed antibiotics [27]. Beta-lactams, particularly third-generation cephalosporins and amoxicillin, are broad-spectrum antibiotics commonly used as first-line treatments for common bacterial infections during hospitalization (urinary, respiratory, digestive, neurological infections, etc.) [28]. They are included in most national and international antibiotic therapy guidelines as they are on the WHO model list of essential medicines [29].

Ceftriaxone also has a long half-life (6–9 hours), allowing for once-daily administration, which could reduce the workload of healthcare personnel in the African context, where human resources are limited.

The frequent use of metronidazole could be explained by the fact that it is one of the few antibiotics effective against anaerobic bacteria, which are typically responsible for intra-abdominal and gynecological infections and abscesses, as well as its lower cost [30].

### 5.4 Microbiological documentation of infections

This survey revealed that 73.4% of curative antibiotic treatments were empirical. In most sub-Saharan African studies, antibiotic prescriptions are not based on microbiological documentation. In Benin, Gnimavo et al. reported that 94.5% of antibiotic prescriptions lacked bacteriological evidence [12]. In Amponsah’s survey in Ghana, most prescriptions were empirical, as only 2.7% of cases had microbiological samples taken for bacterial testing [15]. However, in surveys from developed countries, such as France, where 46.8% of prescriptions were documented [20], a large portion of bacterial infections in hospitals are treated with antibiotics on the basis of microbiological evidence. While not all bacterial infections require microbiological documentation, these results reflect the technical limitations in our regions, which do not always allow identification of the bacteria responsible for the treated infections. Moreover, in patients who have taken antibiotics in community settings before admission to the hospital, it is more difficult to isolate pathogens. However, since this was a one-day cross-sectional survey, there were bacterial infections where microbiological testing was still in progress on the day of the survey. The high rate of empirical treatment may contribute to therapeutic failure in some patients due to inappropriate microbiological targeting. It may also have ecological consequences by exerting selective pressure on bacteria.

### 5.5 Guidelines compliance

In this study, 62% of the antibiotic prescriptions adhered to local antibiotic therapy guidelines. This result is similar to that reported in the multicentric survey by Alfandari et al. in France, which reported 62% compliance rates [20], but higher compliance rates were reported in Uganda (67%), Ghana (84%), and Tanzania (88%) [31]. Aboderin et al. in Nigeria [25] and Chizimu et al. in Zambia [32] reported lower compliance rates, with proportions of 39.4% and 53%, respectively. This discrepancy in results from the literature could be related to the lack of objective and standardized criteria to define good compliance with antibiotic therapy guidelines. The method of evaluating antibiotic prescription quality can vary from study to study. These differences could also be linked to antibiotic stewardship policies, which vary from country to country, or the unavailability of certain first-line antibiotics in some healthcare settings, leading prescribers to use inadequate protocols. However, the antibiotic stewardship activities conducted by the MAT at Dakar Principal Hospital could partly explain the higher compliance rate with local guidelines compared with other facilities.

### 5.6 Use of antibiotics from the watch group

Antibiotics from the watch group were prescribed to 54.4% of the patients included in this survey, with particularly high use among newborns and ICU patients. These antibiotics accounted for 40.5% of all antibiotic prescriptions. Discordant results are often reported in the literature, although significant use of antibiotics from the Watch group seems to be more commonly reported in our regions. A global PPS covering 69 countries across all continents revealed that the use of antibiotics from this group varied from 41.3% to 66.1% [33]. In Bangladesh, 64% use of antibiotics from the Watch group was reported [34]. In the study by Gnimavo et al. in Benin, 69.6% of the patients included had received antibiotics from the Watch group [12]. All these results deviate from the WHO’s 2024--2029 AMR Action Plan, which aims for 70% use of antibiotics from the Access category across the entire healthcare system [11]. The multivariate analysis of factors explaining the use of antibiotics in the Watch group revealed that these antibiotics were 12 times more commonly prescribed in patients with healthcare-associated infections. This is explained by the fact that healthcare-associated infections are often caused by multidrug-resistant bacteria, which are only susceptible to broad-spectrum antibiotics [35].

### 5.7 Scientific implications of the research

This Point Prevalence Survey (PPS), rarely conducted in French-speaking Africa, provides a snapshot assessment of antibiotic use among hospitalized patients at Dakar Principal Hospital. However, it also opens avenues for improvement in both practical applications and research.

- Practical implications:

This study highlights the need to implement antimicrobial stewardship programs in healthcare facilities, as recommended by the WHO, to enhance antibiotic prescribing practices. It would also be beneficial to integrate the AWaRe classification into the revision of the national essential drug list to better protect certain classes of antibiotics. Antibiotic stewardship teams in healthcare facilities should also rely on this classification to regulate broad-spectrum antibiotic prescriptions. Given the high consumption of antibiotics from watch group for healthcare-associated infections, robust infection prevention and control policies should be a priority in tertiary care hospitals across Africa. In addition, several measures should be implemented to reduce the high rate of empirical antibiotic use:

- improving laboratory capacity to enable accurate microbiological diagnosis
- strengthening communication between microbiologists and physicians to enhance sample quality and reduce turnaround time for results
- ensuring microbiological samples are collected before initiating antibiotic therapy.
- Research implications:

This type of survey should be conducted regularly in healthcare settings to monitor trends in antibiotic use and assess the impact of implemented interventions. The integration of pharmaco-epidemiological data would facilitate the surveillance of the most frequently used antibiotic classes and those most implicated in the emergence of resistance. PPS studies also provide opportunities to evaluate hospital antibiotic stewardship policies and estimate the prevalence of nosocomial infections. In the African healthcare context, it would be useful to include outpatients in antibiotic prescription evaluations.

Additionally, qualitative and mixed-method studies should be conducted to better understand the barriers to noncompliance with local guidelines and propose effective improvement strategies.

### 5.8 Strengths and limitations of the study

Several strengths of this study should be noted. It was conducted following a methodology validated by the WHO and adopted worldwide. It then provided epidemiologically relevant results comparable to available antibiotic use data in the literature. The medical files were also well held in the different wards, allowing the collection of quasicomplete data from the included patients. The analytical component performed to determine the factors explaining the use of broad-spectrum antibiotics provides insights into the fight against their overprescription. However, this study has limitations. First, it was a cross-sectional study conducted on a single day, which does not allow for continuous and exhaustive evaluation of antibiotic prescriptions. Second, the study was conducted in a single healthcare facility with a relatively small sample size, which could reduce the statistical power of the analyses and prevent extrapolation of the results to other hospitals. Finally, five eligible patients were not included because their medical records were unavailable on the day of the survey.

## 6. Conclusion

This study revealed frequent antibiotic use at the Principal Hospital of Dakar, with an uneven distribution across departments. Compliance with local recommendations is relatively good, despite the low rate of microbiological documentation for infections treated with antibiotics. This study also highlights the significant use of antibiotics from the watch group, especially in healthcare-associated infections. Therefore, strengthening antibiotic prescription policies and measures to prevent healthcare-associated infections is necessary for more rational antibiotic use. Periodic multicentric prevalence surveys at the national level are needed for proper monitoring of antibiotic use in healthcare facilities.
